# The Treatment of Prostatic Hypertrophy

**Published:** 1898-01

**Authors:** C. Ferdinand Durand

**Affiliations:** Buffalo, N. Y.


### THE TREATMENT OF PROSTATIC HYPERTROPHY.

BY C. FERDINAND DURAND, B. A., M. B., Buffalo, N. Y.

PROSTATIC hypertrophy is said to be present in 33 per cent. of men over 55 years of age and in about 10 per cent. to be of pathological importance. In view of its usually insidious onset and progressive character, and from the fact that its early recognition is of the first importance, a review of the methods adopted for its treatment may not be out of place. Under the term "prostatic hypertrophy" several more or less distinct pathological conditions may be included. There may be uniform passive congestion or subacute inflammation of the entire gland, hyperplasia of the interstitial, muscular or glandular elements or distinct fibromyomata or fibroadenomata may be present.

It is the duty of the general practitioner to constantly bear in mind this condition and to warn those of his patients likely to be affected by it to inform him of any frequency of urination with which they may be troubled, so that by proper advice they may take those hygienic and dietetic precautions indicated in such cases. When men past middle age have to urinate twice during the night, and especially when the force of the stream is decreased, other pathological conditions being absent, it is an indication that the prostate may be hypertrophied.

Such men should be warned to avoid getting chilled, delaying to obey the call to urinate and excesses in eating, drinking and venery; they should keep the bowels regular, wear warm underclothing and attend to the functions of the skin.

When there are no other symptoms pointing to urinary difficulty, such treatment is likely to prove sufficient and retention will probably not occur. When, however, men of this age are obliged to urinate three or more times during the night and more often than formerly during the day, and when there is in addition some tenesmus and enfeeblement in the force of the stream, it is probable that there is some retention and they should be aseptically catheterised to determine the amount of the residual urine. When this does not exceed one or two ounces and there is no cystitis nor other pathological condition present and catheterisation is well borne, using the catheter at bedtime may be sufficient.

There are also many cases where the residual urine greatly exceeds this amount, but where cystitis is absent, in which more frequent catheterisation seems to act well. In all cases, however, when catheterisation is badly borne, when cystitis or distension of the bladder occurs, some operative measure should be undertaken and as speedily as possible.

In all such patients it is important to avoid catheterisation, which often causes a series of pathological conditions that ends in pyelo-nephritis and death.

When it has been decided to open the bladder, the suprapubic operation should, I think, be selected, as it offers exploratory advantages that the perineal operation does not afford. When the bladder is opened above the pubes the obstruction can be both felt and seen, its exact character ascertained and the necessity and kind of radical operation determined. The double operation of Belfield, who opens the bladder both above the pubes and through the perineum, is of advantage in certain cases.

Many times all that is necessary is to treat the cystitis present and afford constant drainage by having the patient wear a suitable tube, for, in course of time, the inflammation of the prostate subsides and natural urination is restored. There is no contraindication to suprapubic cystotomy, unless it be a very much contracted bladder, and the more desperate the condition of the patient the more urgently is the operation indicated. Double orchidectomy, with which the name of White, of Philadelphia, is associated, seems to act well in some instances, but, in addition to its esthetic objection, it is sometimes followed by alarming mental disturbance and considerable mortality.

Vasectomy, as recommended by Guyon, also by Daggett, of Buffalo, has also had some good results, but is only in the experimental stage. It may be that both these operations are of benefit when the obstruction is largely of a glandular nature.

The lateral prostatectomy of Dittel, Bier's method of tying both internal iliac arteries and the electro-caustic gauging operation of Bottini have found little favor in this country.

The point I wish to emphasise is the necessity of operating early. When catheterisation is for any reason badly borne, when cystitis or distension of the bladder is present, no temporising measures should be adopted, but suprapubic cystotomy should be performed.

258 FRANKLIN STREET.

